# Surface Oxidation of Transition Metal Nitrides

**DOI:** 10.1021/acs.jpcc.5c02303

**Published:** 2025-06-06

**Authors:** Ji Liu, Jean-Pierre Glauber, Julian Lorenz, Detlef Rogalla, Corinna Harms, Anjana Devi, Michael Nolan

**Affiliations:** a 261183 Tyndall National Institute, University College Cork, Lee Maltings, Dyke Parade, Cork T12 R5CP, Ireland; b 28394 Leibniz Institute for Solid State and Materials Research, Helmholtzstr. 20, Dresden 01069, Germany; c Inorganic Materials Chemistry, 9142 Ruhr University Bochum, Universitätsstraße 150, Bochum 44801, Germany; d Institute of Engineering Thermodynamics, German Aerospace Center (DLR), Carl-von-Ossietzky-Str. 15, Oldenburg 26129, Germany; e RUBION, Ruhr University Bochum, Universitätsstr. 150, Bochum 44801, Germany; f Fraunhofer Institute for Microelectronic Circuits and Systems, Finken Str. 61, Duisburg 47057, Germany; g Chair of Materials Chemistry, Dresden University of Technology, Bergstr. 66, Dresden 01069, Germany

## Abstract

The well-known Haber–Bosch process for NH_3_ production is highly inefficient, with a significant energy demand and CO_2_ emissions. Alternative approaches, including electrochemical ammonia synthesis from N_2_ and H_2_, are attractive, but the sluggish nitrogen reduction reaction (NRR) that arises from the high energy input to activate N_2_ remains a significant challenge for NRR electrocatalysis. The nitrogen-rich surface of transition metal nitrides (TMNs) can deliver one solution to this challenge. A Mars–van Krevelen-like mechanism is proposed that forms N vacancies via hydrogenation and ammonia release, followed by vacancy filling through N_2_ activation. We recently showed that ZrN thin films deposited with metal–organic chemical vapor deposition (MOCVD) are rapidly oxidized when exposed to ambient conditions during *ex situ* handling prior to analysis and showed preliminary results, from *ab* initio molecular dynamics (aiMD) simulations, indicating that surface oxidation is favorable. In this paper, we investigate in detail with aiMD the unintentional oxidation of ZrN and VN surfaces by oxygen present at ambient conditions at various temperatures: 295, 363, 873, and 1023 K. Results show that ZrN surfaces tend to form oxynitrides at lower temperatures and prefer to form a ZrO_*x*_ layer interfaced with ZrN at higher temperature. By contrast, VN(111) forms VO_*x*_ clusters on the surface, and there is no significant migration of the O species into bulk VN at all studied temperatures. We attribute the different oxidation processes of ZrN and VN to the relative strengths of V–N/O bonds and Zr–O/N bonds—the bond dissociation energy of V–N (452 kJ/mol) is larger than that of Zr–N (339 kJ/mol), while the V–O bond (645 kJ/mol) is weaker than the Zr–O bond (776 kJ/mol). Experimental results on MOCVD nitride films, including Rutherford backscattering spectrometry in combination with nuclear reaction analysis (RBS/NRA), confirm that VN is less oxidized than ZrN at ambient conditions because VN forms a less stable, potentially volatile oxide layer, whereas ZrN has a stronger tendency to form a stable, protective ZrO_2_ layer, promoting more complete oxidation at higher temperatures. This study defines a new degree of atomic-scale understanding of the formation of oxynitride or separated oxide phase in TMNs at ambient oxygen conditions relevant for NRR electrocatalysis.

## Graphical abstract

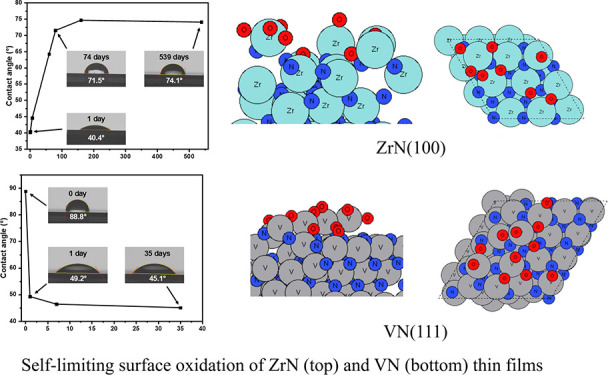

## 1 Introduction

Ammonia (NH_3_) plays a truly enormous role in the modern world. Most of the world’s production of ammonia goes to fertilizer for crops that feed almost half of the global population, with the remainder used in plastics, explosives, fabrics, and other materials.^1^ Made of nitrogen and hydrogen, ammonia is also an important carbon-free energy carrier and makes an appealing sustainable fuel, since it burns without releasing carbon. The major issue lies in the fact that NH_3_ production by the Haber–Bosch process is highly inefficient. According to some estimates, it contributes to ca. 2% of global energy demand and to 1% of total annual global emissions of greenhouse gases through the release of more than 400 million tons of CO_2_ per year.^2^ There is an urgent need for new technologies that can synthesize NH_3_ in a sustainable and carbon-neutral way. The heterogeneous electrochemical nitrogen reduction reaction (NRR) is promising as it uses renewable energy-powered water electrolysis as a proton source.^3–5^

Transition metal nitrides (TMNs) are interstitial alloys that incorporate nitrogen atoms into the interstitial sites of the crystal lattice of a transition metal. Unique properties that make TMNs attractive include brittleness, high electrical and thermal conductivity similar to metals.^6^ In addition, TMNs are ultrahard and have high melting points like covalent solids and take simple rock-salt structures like ionic solids.^7^ Thus, TMNs have been applied in different fields. For example, conductive TiN and TaN are used in microelectronic circuits due to their excellent properties as Cu diffusion barrier materials.^8^ TMNs are also known as catalytically active materials for several reactions, including NH_3_ synthesis,^9^ CO oxidation,^10^ and NO reduction.^11^

Most importantly, TMNs are attracting increasing attention as a new class of nonprecious metal catalysts for NRR,^12^ utilizing the N-rich terminations to promote the otherwise sluggish kinetics via a Mars–van Krevelen (*MvK*) mechanism.^13,14^ In this mechanism, the N species in the nitride is electrochemically reduced to NH_3_ under electrochemical NRR conditions, and the resulting N vacancy is then recovered with gaseous N_2_ to regenerate the metal nitride catalyst surface. One key benefit of this *MvK* mechanism is that sluggish NRR is overcome by suppressing the competing HER and reducing the high energy required to break the triple bond in the N_2_ molecule.^15^

Previous studies^16,17^ have pointed out that candidates such as ZrN and VN could be more selective toward NRR than HER. For example, the rock-salt structured ZrN (100) and VN (100) facets^18^ are theoretically described as possibly active and selective for the NRR and stable under typical electrocatalytic potentials. Nevertheless, there are some controversial experimental studies on the catalytic ability of TMNs toward NRR, questioning the limited activity and stability of bulk TMNs and demonstrating noncatalytic decomposition of the catalyst instead of genuine NRR activity.^19^ Thus, understanding the surface structure and its properties is important to improve the catalytic ability of TMNs toward NRR.

It is predicted that when metal nitrides are taken for *ex situ* characterization, their handling in ambient conditions means that oxygen will react at the nitride surface to form transition metal oxynitrides (TMONs).^20,21^ An advantage of TMONs is the tunability of the N:O composition and thus their catalytic properties, which then alter the mechanistic pathways for NRR.^22^ For example, partially formed oxynitrides of VN,^23^ CrN,^24^ and NbN^25^ have been experimentally described as the active species for NRR. Therefore, understanding the effect of oxygen incorporation into the TMN surface and tailoring the catalyst surface morphology to promote high efficiency on a large scale are crucial for implementation of TMNs in practical catalysis and remain a grand challenge in terms of directed synthesis and rigorous experimental NRR evaluation. The latter is prone to false-positive results due to very low production rates and Faradaic efficiencies as well as multiple contaminants in electrochemical NRR experiments.^26^ Therefore, NRR data for catalysts in aqueous electrolytes must be evaluated with caution. Although final proof by isotope labeling experiments was not achieved due to a lack of sufficient concentration for NMR analysis, promising experimental results were reported for sputtered ZrN films.^27^ By contrast, VN and NbN both produce ammonia by releasing their N atoms at the beginning of electrochemical experiments, which then become inactive by depleting their surface N atoms.^27^

Recently, we have demonstrated the synthesis and potential of nanoparticulate ZrN powder materials^28^ and ZrN thin films^29^ through qualitative NRR experiments. The importance of controlling the oxygen content and the literature reports on the activity of oxynitrides,^21,24,25^ potentially enhancing the NRR activity, motivate us to study the surface oxidation of TMNs in more detail using atomistic simulations and thin film synthesis.

With this idea in mind, the growth process of TMNs requires an in-depth study and analysis. Tailoring the catalyst surface morphology toward high-efficiency NRR on a large scale is crucial for implementation of TMNs in practical catalysis and remains a grand challenge in terms of experimental fabrication and growth development.^9^ These grand challenges motivate the use of metal–organic chemical vapor deposition (MOCVD) as the method of choice for metal nitride thin film deposition since it allows precise tuning of the surface features and the composition of the material by variation of the process parameters including the precursor, temperature, and pressure.^30^ In addition, MOCVD is industry-compatible because it enables scalability of catalyst production toward high volume. In a recent publication, we have presented a detailed study on the decomposition mechanism of a single-source MOCVD precursor (SSP) and the analysis and characterization of deposited ZrN thin films using MOCVD.^29^ A mixed amide and guanidinate precursor, namely, [Zr­{η^2^(*i*PrN)_2_CNMe_2_}_2_(NMe_2_)_2_], i.e., [Zr­(guan)_2_(NMe_2_)_2_], was synthesized and successfully employed in SSP MOCVD of ZrN on Si and glassy carbon (GC) substrates at moderate process conditions. The ZrN thin film was deposited, characterized, and analyzed with complementary analytical methods including X-ray diffraction (XRD) and X-ray photoelectron spectroscopy (XPS). Bulk compositional analysis by Rutherford backscattering spectrometry (RBS) coupled with nuclear reaction analysis (NRA) revealed slightly nitrogen-rich films with some oxygen incorporation (measured at 19 at. %), while surface-sensitive XPS suggested a Zr oxide surface termination in addition to minor oxynitride and nitride components. Oxygen contamination is excluded during the MOCVD process, and the oxygen incorporation is therefore attributed to the postdeposition handling and processing at ambient conditions with direct contact to air, which has implications for the handling and storage of TMNs.

Given that metal nitrides form oxynitride^21,31^ and these are potentially effective for NRR, it is important to study the initial steps of the oxidation of TMNs, where theorical simulations can deliver insights to understand the initial oxidation process at the atomic scale.^32–35^ Previous theoretical studies of initial steps in the oxidation of early TMNs have found out that the initial adsorption and dissociation of O_2_ are always exothermic on TMNs such as TiN and VN, and DFT relaxations have shown that incorporation of surface O atoms into the bulk TiN or VN is endothermic.^32^ This work additionally pointed out that there are strong barriers to the diffusion of oxygen atoms into the bulk for these metal nitrides.

In the present study, we go beyond standard DFT relaxations and perform *ab initio* molecular dynamics (aiMD) simulations to study the oxidation process of ZrN and VN surfaces at selected temperatures, for which the initial results highlight that oxidation of the terminal ZrN surface is favorable,^29^ while the synthesis of VN is currently ongoing. We compare our results to a previous study^29^ for ZrN, and preliminary data for oxidation of MOCVD-grown VN are presented herein. A detailed study of VN MOCVD, characterization, and NRR testing is the subject of a separate publication.

Our general simulation approach is to introduce multiple oxygen species and run aiMD simulations as follows: We start with oxygen atoms positioned above the nitride surface, run the dynamics, and inspect the structure at the end of this simulation, removing from the supercell any oxygen that did not bind, form O_2_, or form free NO_*x*_. A new set of oxygen atoms are added, and these steps are repeated until, upon addition of a new set of O species, the resulting structure has reached a saturation coverage of oxygen and no further oxygen incorporation can take place. This structure is then relaxed. The major result is that although oxygen can incorporate into both TMNs, on ZrN, a ZrON layer forms at lower temperatures, which transitions into a ZrO_2_ layer interfaced with ZrN at higher temperatures, while VN only undergoes some surface oxidation, with no oxygen migration toward the bulk and only a thin VO_*x*_ layer forms. Experimental results confirm the difference between the two TMNs. This study, bringing together aiMD and experiments on the same materials, defines new atomic-scale understanding on the formation of the oxynitride phase or separated oxide phase at ambient oxygen conditions relevant for NRR electrocatalysis.

## 2 Computational Details and Experimental Methods

### 2.1 Computational Details

All the calculations are performed on the basis of periodic spin-polarized density functional theory (DFT) within a plane wave basis set and projector augmented wave (PAW) formalism,^36^ as implemented in the Vienna *ab* initio simulation package (VASP 5.4) code. The generalized gradient approximation (GGA) with the parametrization of Perdew–Burke–Ernzerhof (PBE) is used for the exchange-correlation functional.^37,38^ We use 12 valence electrons for Zr, 5 for V and N, and 6 for O. The plane wave energy cutoff is set to be 400 eV. The convergence of energy and forces are set at 1 × 10^–4^ eV and 1 × 10^–3^ eV/Å, respectively. The unit cell of ZrN has a computed lattice constant of *a* = *b* = *c* = 3.26 Å and angles of α = β = γ = 60°, whereas the unit cell of VN has a computed lattice constant of *a* = *b* = *c* = 4.29 Å and angles of α = β = γ = 90°.

To simulate the oxidation of ZrN and VN thin films by oxygen present in ambient *ex situ* conditions (the present simulations cannot model the ambient atmosphere as we do not control pressure or include other species so that the focus is on the role of oxygen that is present in ambient conditions on the nitride surface oxidation), aiMD calculations were performed in the NVT (canonical) ensemble with VASP 5.4 at selected temperatures to assess the impact of different temperature regimes on the extent of oxidation. These temperatures are 295 and 363 K since samples are generally handled post growth at temperatures in this range, while the higher temperatures are 873 and 1023 K, corresponding to the respective growth temperatures of VN (111) and ZrN (200).

We are exploring the use of MOCVD to promote facet-controlled synthesis of metal nitrides, as this is another way to tune the NRR activity. In this paper, we investigate various facets of ZrN and VN, focusing on ZrN (100), ZrN (110), and VN (111). For ZrN, this comparison allows the impact of the surface facet on oxidation to be explored. Among these facets, ZrN (100) and VN (111) are the dominating facets from the XRD analysis of deposited ZrN previously published^29^ and VN thin films that are currently being optimized experimentally. Slab thicknesses of 24.33 and 23.67 Å are used to model ZrN and VN films, a (3 × 3) supercell is applied for ZrN (100) and ZrN (110) facets, and a (4 × 4) supercell is applied for the VN (111) facet. The configurations of all the slab models are shown in Figure 1. For ZrN (100), Zr atoms and N atoms are located alternatively in each layer, resulting in 9 Zr atoms or 9 N atoms in each layer, while for ZrN (110), Zr atoms and N atoms are in the same layer, resulting in 18 atoms in total in each layer. The VN (111) slab model shares a similar atom orientation to the ZrN (100) slab, where V atoms and N atoms are located alternatively in each layer, resulting in 16 V atoms and 16 N atoms in each layer.

**Figure 1:**
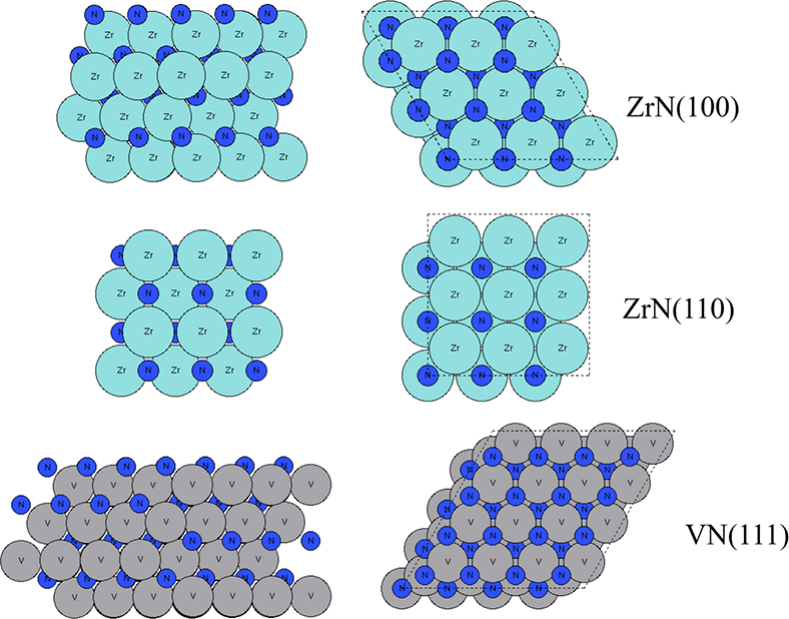
Atomic structures in top and side views, showing the first layers, of the slab models of (top to bottom) ZrN(100), ZrN(110), and VN(111). Zr atoms and N atoms are presented in light blue and dark blue, respectively, and V atoms are presented in gray color.

A vacuum region up to 15 Å is applied for all of the slab models. A *k*-point mesh^39^ at 3 × 3 × 1 was used for all the aiMD calculations and structure relaxation calculations after aiMD simulations. The time step was 2.5 fs, with a total running time of 6.25 ps.

The aiMD calculations are performed as follows: we introduce 6 oxygen atoms, corresponding to 3 dissociated O_2_ molecules, above ZrN (100) and ZrN (110) surfaces at random locations in the supercell, while for VN (111), the larger surface supercell area means that we introduce 10 oxygen atoms above the surface in the first aiMD calculation. After each aiMD calculation, the free O species, recombined O_2_ species, and other byproducts, e.g., NO and N_2_, are removed before proceeding to the next round of aiMD calculations, in which 6 O atoms or 10 O atoms are positioned above the surface for ZrN and VN, respectively. Here, we use ZrO_*x*_/ZrN and VO_*x*_/VN to represent the resulting ZrN and VN thin films after the incorporation of O incorporation. We identify the formation of oxynitride phases or a separated oxide phase via structure relaxations that start from the final aiMD structure for each simulation.

### 2.2 Experimental Methods

#### 2.2.1 Thin Film Deposition

The depositions of ZrN and VN thin films on Si substrates were carried out in a custom-built horizontal cold-wall CVD reactor employing SSP [Zr­(guan)_2_(NMe_2_)_2_] and a newly developed vanadium precursor, respectively. The ZrN precursor was evaporated at 135 °C, while the vanadium precursor was evaporated at 110 °C. For both target materials, nitrogen (5.0) was employed as carrier gas and VN deposition was carried out in the presence of dry ammonia. Prior to deposition, Si(100) (1 × 1 cm^2^, p-type) substrates were cleaned consecutively with HPLC-grade acetone, isopropanol, and water in an ultrasonic bath, followed by drying with argon (5.0). ZrN was deposited by employing the optimized conditions: 100 sccm N_2_ carrier gas flow at a temperature of 750 °C at 1 mbar. For VN, a N_2_ carrier gas flow of 25 sccm, an ammonia flow of 20 sccm, a deposition temperature of 550 °C, and a deposition pressure of 10 mbar were employed.

#### 2.2.2 Thin Film Characterization

XRD was measured using a Bruker D8 advance diffractometer with Cu–Kα radiation (λ = 1.5418 nm) in Bragg–Brentano (θ–2θ) geometry over a range of 20–60° for ZrN and 20–65° for VN. The applied acceleration voltage was set to 60 kV, and the heating current was 30 mA. RBS and NRA measurements were performed at a 4 MV tandem accelerator of the RUBION facility (Ruhr University Bochum, Germany). For RBS, a 2 MeV ^4^He^+^ beam (intensity 40–50 nA) incident to the sample at a tilt angle of 7° was used. NRA analysis was performed by using a 1 MeV deuteron beam at an equal tilt angle. The emitted protons were detected with a silicon detector. The backscattered particles were measured at an angle of 160° using a silicon detector with a resolution of 16 keV. The stoichiometry was calculated from the RBS and NRA data using the program SIMNRA.^40^

## 3 Results and Discussion

### 3.1 Oxidation Step of ZrN(100)

We first present the aiMD results of oxidation on the ZrN (100) surface at 295, 363, and 1023 K, as explained in the previous section. For *T* = 295 K, the aiMD results show that in total, three rounds of addition of the O atoms (in total 18 oxygen atoms) could be performed, resulting in self-limiting oxidation. The configurations of ZrO_*x*_/ZrN after each aiMD round are shown in Figure 2. After aiMD simulation with the first 6 O species, two oxygen atoms bind to surface Zr and N atoms, and one Zr atom is shifted upward by 1.78 Å along the *z*-axis. After introducing a further 6 O atoms in the aiMD simulation, two more oxygen atoms bind to the surface Zr atoms, and two more Zr atoms are shifted upward by 1.75 and 1.93 Å along the *z*-axis. The Zr atoms that migrated upward form the new outmost layer. After adding a third set of 6 O atoms to the aiMD simulation, we clearly see the presence of a Zr–O4 coordination with Zr–O distances in the range of 1.91–2.24 Å and a maximum of 8 oxygen atoms can be introduced to oxidize the surface ZrN, forming a Zr_4_O_7_ terminal layer with an additional O atom binding to a subsurface Zr atom.

**Figure 2:**
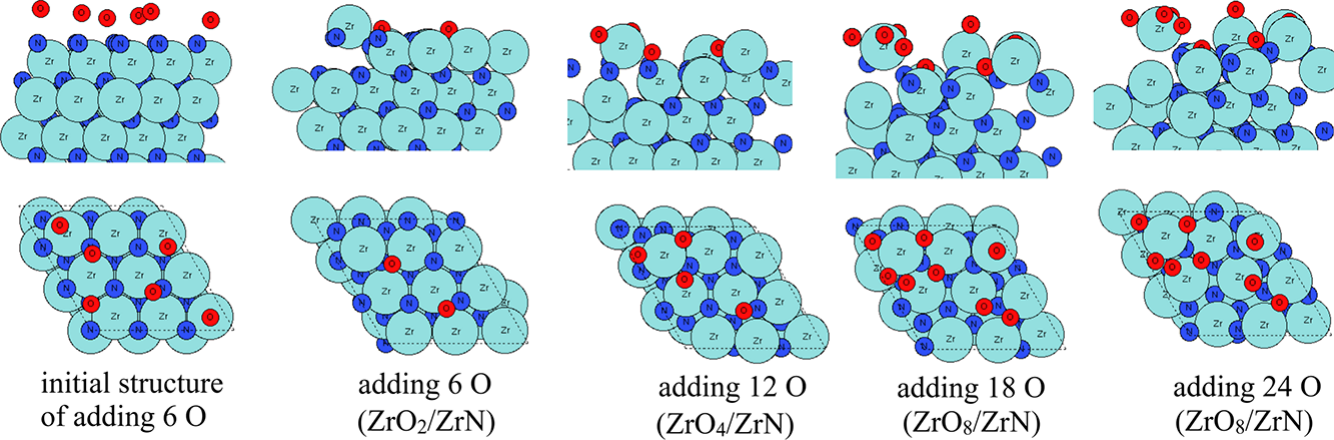
Atomic configurations after aiMD of ZrN surface oxidation on the ZrN (100) surface at room temperature (295 K). Zr atoms and N atoms are presented in light blue and dark blue colors, respectively, while oxygen atoms are presented in red color.

When more oxygen is added to the aiMD simulation, these added oxygen atoms cannot bind to the surface and desorb from the surface through recombination to O_2_. At room temperature, the oxidized Zr atoms are mostly present in the surface region, indicating that a mixed layer of oxynitride, which is terminated with Zr–O, is formed in the surface region.

For *T* = 363 K, the configurations resulting from the aiMD simulations are shown in Figure 3. Here, the starting configuration is the same structure as shown in Figure 2, so it is omitted in Figure 3. After aiMD simulation with the first 6 O species, four oxygen atoms bind to surface Zr and N atoms, and two Zr atoms are shifted upward by 1.66 and 1.56 Å along the *z*-axis. After introducing a further 6 O atoms in the aiMD simulation, two more oxygen atoms bind to the surface Zr atoms, and two more Zr atoms are shifted upward by 1.75 and 1.56 Å along the *z*-axis. After introducing 30 O atoms (5 successive aiMD calculations), six Zr atoms migrate upward to form a new terminal layer, and at most 12 O atoms are present at the surface and subsurface region. Zr–O_4_ coordination and Zr–O_2_ coordination with Zr–O distances in the range of 1.99–2.31 Å are clearly observed after the fifth round of adding 6 O atoms in the aiMD simulation. When more oxygen is added to the aiMD simulation, the surface oxygen atoms change their sites, but no new added O atoms bind to the ZrN substrate, resulting in a ZrO_12_/ZrN composition at 363 K. Compared to ZrO_*x*_/ZrN formed at room temperature, at 363 K, more Zr atoms are shifted upward to form Zr–O bonds, and we see the tendency to form a separate ZrO_2_ layer in the form of Zr_6_O_11_ in the outermost layer. Some introduced oxygen migrates to the subsurface layer, and this indicates that increasing the temperature promotes the inward migration of O species into the subsurface ZrN layer, which supports the transition to the formation of a separate ZrO_2_ layer at higher temperatures.

**Figure 3:**
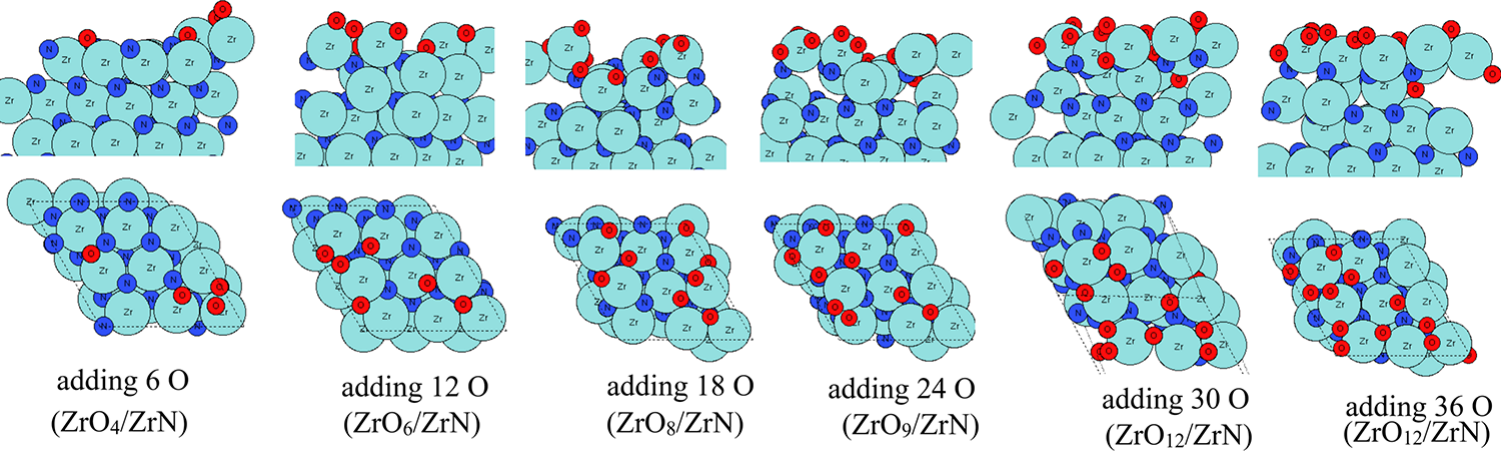
Atomic configurations after aiMD of ZrN surface oxidation on the ZrN (100) surface at 363 K. Zr atoms and N atoms are presented in light blue and dark blue colors, respectively, and oxygen atoms are presented in red color.

To understand the possible high-temperature oxidation of ZrN (100), we perform the aiMD calculations at 1023 K. At such a high temperature, significant Zr migration occurs, and the surface region of ZrN (100) is highly distorted to form a clear ZrO_*x*_ layer. The configurations of the resulting structure after adding 6 atoms of O in each successive aiMD simulation step are shown in Figure 4. Here, the starting configuration is the same structure as that shown in Figure 2. Starting from the first aiMD calculation with 6 O species, we clearly see the migration of O atoms into the subsurface region and gradually into bulk ZrN. N atoms in the surface layers, for example, first, second, and third layers, are removed via N_2_ and NO formation. A clear ZrO_2_ layer is formed after the eighth round of adding 6 O atoms (adding 48 in total) in the aiMD calculations, where the surface region of ZrN is fully oxidized to a ZrO_2_ layer. The resulting structure includes a fully oxidized surface region of Zr_11_O_22_ (first three layers), a mixed layer of oxynitride, and a ZrN substrate. We stop after adding 48 oxygen atoms in the aiMD runs because a distinct ZrO_2_ layer is formed. We can conclude that the surface region of ZrN is sensitive to temperature together with exposure to oxygen, where it could be easily oxidized to a separate ZrO_2_ layer rather than a mixed oxynitride layer. The extent of surface oxidation is highly relevant for the understanding of surface-driven electrocatalytic NRR and subsequently relevant for improving experimental catalyst materials.

**Figure 4:**
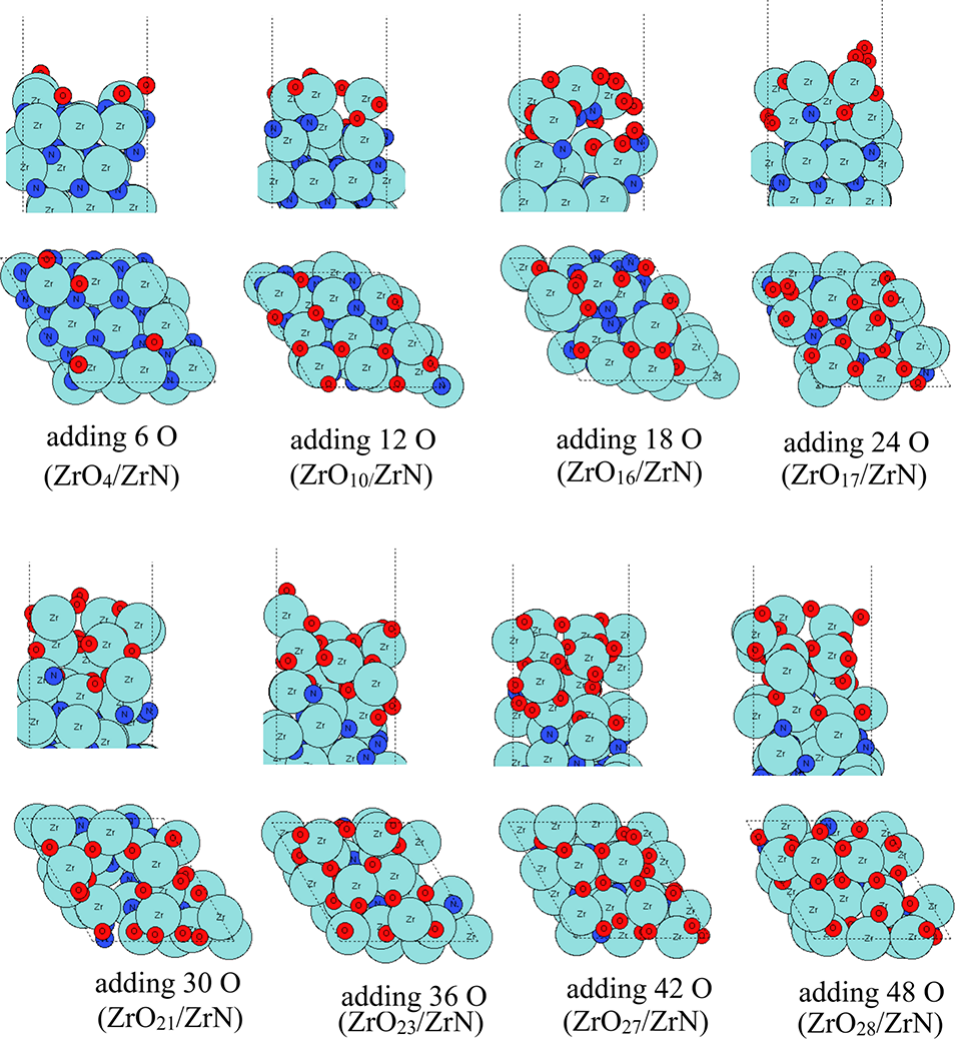
Atomic configurations after aiMD of ZrN surface oxidation on the ZrN (100) surface at 1023 K. Zr atoms and N atoms are presented in light blue and dark blue colors, respectively, and oxygen atoms are presented in red color.

### 3.2 Oxidation of ZrN (110)

Given that our synthesis efforts aim to produce facet-controlled metal nitrides for NRR, this section discusses the effect of the surface facet orientation on the oxidation process. For the ZrN (110) substrate, we perform the aiMD calculations at 295, 363, and 1023 K.

For *T* = 295 K, the configurations after the addition of a total of 36 O atoms in the aiMD calculations are shown in Figure 5. In the first three aiMD runs (each run adding 6 O atoms), all the newly added O atoms bind to the ZrN (110) surface and can migrate into the subsurface region, showing a significant difference already in the oxidation process compared with ZrN (100). These O atoms form Zr–O bonds, and Zr atoms migrate to form a new outermost Zr–O layer. When 30 O atoms are introduced, fewer available Zr atoms in the surface mean that newly added O atoms tend to form Zr–O–O-Zr bonds to O atoms available on the surface. After adding 36 O atoms, layers of O species bound to O atoms are formed on the surface, blocking any newly added oxygen from migrating into bulk ZrN (110). These formed O layers can be easily desorbed via increasing the temperature. Thus, we take the structure after adding 24 O atoms as our final structure at room temperature, where the resulting structure includes a surface oxidized Zr_9_O_18_ layer and a ZrN substrate layer, indicating a higher degree of oxidation compared with the (111) surface. The local atomic structure of the (110) surface is more open, typical of higher energy surface facets, which can facilitate migration of O atoms into the subsurface region.

**Figure 5:**
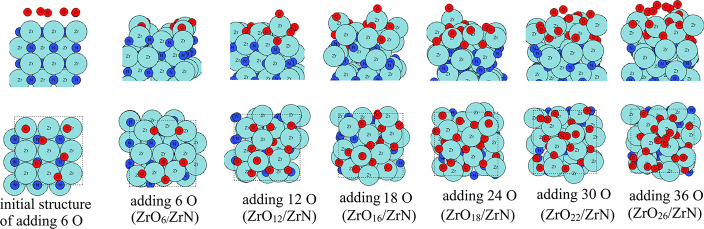
Configurations of the oxidation steps of each aiMD round on the ZrN (110) surface at room temperature (295 K). Zr atoms and N atoms are presented in light blue and dark blue colors, respectively, and oxygen atoms are presented in red color.

The resulting configurations after the aiMD was run at a temperature of 363 K are shown in Figure 6. Analogous to the rapid formation of the surface ZrO_*x*_ layer at 295 K, adding 12 O atoms of aiMD results in upward migration of surface Zr atoms and the tendency to form a ZrO_*x*_ layer in the surface region. After 24 O atoms are added, any subsequent oxygen will not bind to the substrate. The resulting structure includes a surface Zr_10_O_20_ layer and a ZrN substrate layer. This is not observed for ZrN (100) at 295 and 363 K, where a mixed layer of oxynitride layer is formed in the surface region. Additionally, the formed ZrO_2_ layer on ZrN (110) at 295 and 363 K will limit any further oxidation into bulk ZrN, and a self-limiting oxidized layer can be produced.

**Figure 6:**
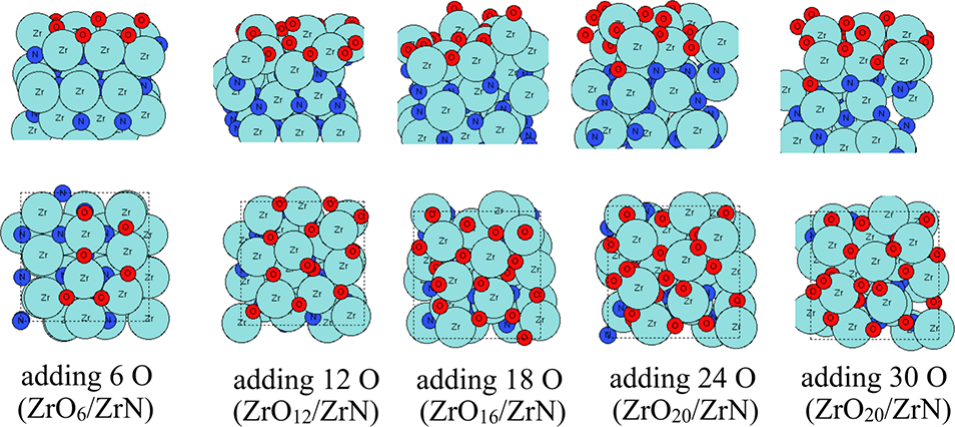
Atomic configurations after aiMD of ZrN surface oxidation on the ZrN (110) surface at 363 K. Zr atoms and N atoms are presented in light blue and dark blue colors, respectively, and oxygen atoms are presented in red color.

At 1023 K, the oxidized ZrO_*x*_ layer is clearly thicker than the ZrO_2_ layer at both room temperature and 363 K. The outmost Zr atoms in the surface (Zr_15_O_30_) are shifted upward to form a new oxide layer. These results are summarized in Figure 7. For a given number of oxygen species added to the model, the number of oxygen atoms incorporated into this ZrN surface is always higher than that on the ZrN (100) surface. If we compare the oxidation process of ZrN (110) at 295, 363, and 1023 K, it is clear that increasing the temperature further promotes the oxidation process, with more Zr atoms in the surface region being oxidized and more subsurface Zr atoms migrating upward to form a new ZrO_*x*_ layer in the outmost region. At 295 K, only the surface Zr atoms are oxidized at the outmost region (Zr_9_O_18_); when we increase the temperature to 363 K, after shifting upward, the first two layers of Zr atoms are oxidized (Zr_10_O_20_); if we increase the temperature to an extreme condition at 1023 K, a surface Zr_15_O_30_ layer is formed, with more Zr atoms being shifted to the outmost region.

**Figure 7:**
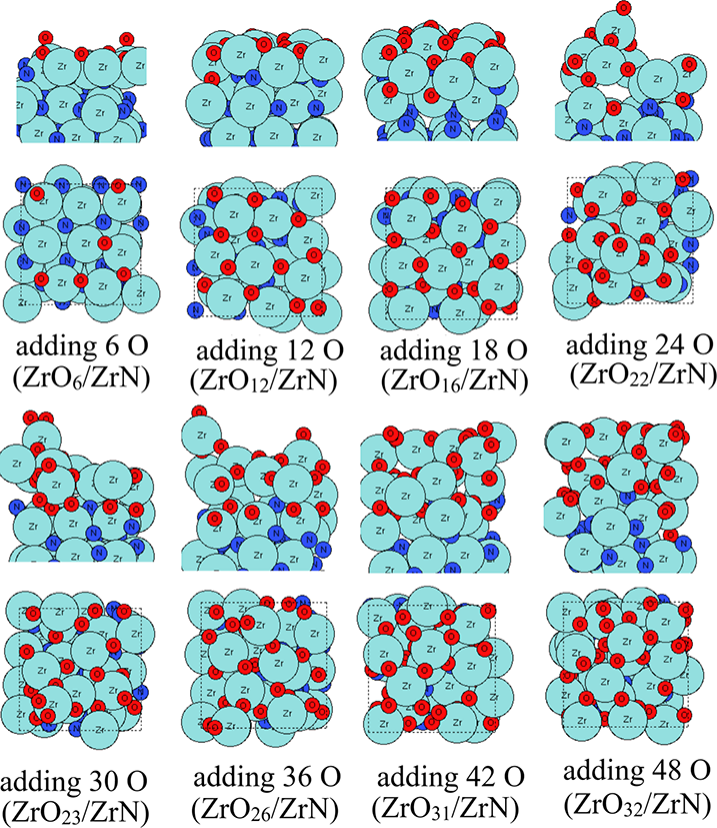
Atomic configurations after aiMD of ZrN surface oxidation on the ZrN (110) surface at 1023 K. Zr atoms and N atoms are presented in light blue and dark blue colors, respectively, and oxygen atoms are presented in red color.

The major difference between ZrN (100) and ZrN (110) is that at room temperature, ZrN (100) tends to form a mixed layer of oxynitride in the outmost region, while a ZrO_2_ layer is formed on the ZrN (110) surface. One reasonable explanation behind this is that ZrN (100) has an alternating distribution of Zr atoms and N atoms in each layer, resulting in a larger Zr–Zr distance (around 3 Å) vertically, and it is therefore more difficult for subsurface Zr atoms to be shifted upward and be oxidized by oxygen species. Meanwhile, for ZrN (110), Zr atoms and N atoms are in the same layer, resulting in a smaller Zr–Zr distance (around 2.1 Å) vertically, and it is easier for Zr atoms to migrate to form the outermost layer and be oxidized to ZrO_2_.

### 3.3 Oxidation of VN (111)

In this section, we investigate the oxidation process of VN (111) at 295, 363, and 873 K to explore the initial oxidation process and compare with ZrN oxidation.

The atomic configurations of VN after the aiMD runs are shown in Figures 8–10 for temperatures of 295, 363, and 873 K, respectively. At room temperature, the oxygen species tend to occupy the outmost surface region and shift some of the surface V atoms upward to form VO_*x*_ on VN substrates. After adding 30 O atoms in the aiMD, no further oxygen species bind to the substrate. On the contrary, we notice that two oxygen atoms that were incorporated in the surface after adding 20 O atoms are now removed via O_2_ formation. The resulting structure includes partially oxidized V_5_O_11_ on top of the VN (111) substrate. Although we simulate VN (111) in a bigger supercell and there are more available V atoms present in the surface, it appears that the shifting upward of V atoms that become oxidized by added oxygen is more difficult than in ZrN (110) and (100), suggesting a less favorable tendency for VN to oxidize compared to ZrN.

**Figure 8:**
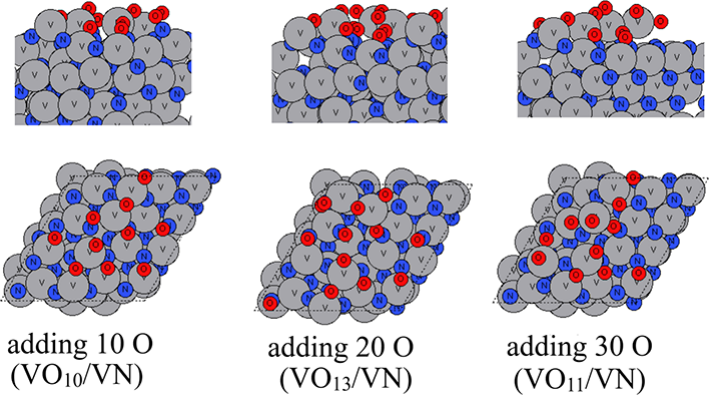
Atomic configurations after aiMD of VN surface oxidation on a VN (111) surface at room temperature (295 K). V atoms and N atoms are presented in gray and dark blue colors, respectively, and oxygen atoms are presented in red color.

**9:**
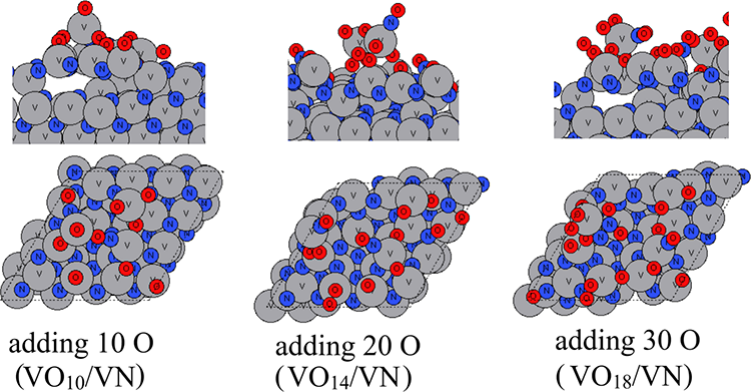
Atomic configurations after aiMD of VN surface oxidation on the VN (111) surface at 363 K. V atoms and N atoms are presented in gray and dark blue colors, respectively, and oxygen atoms are presented in red color.

**Figure 10:**
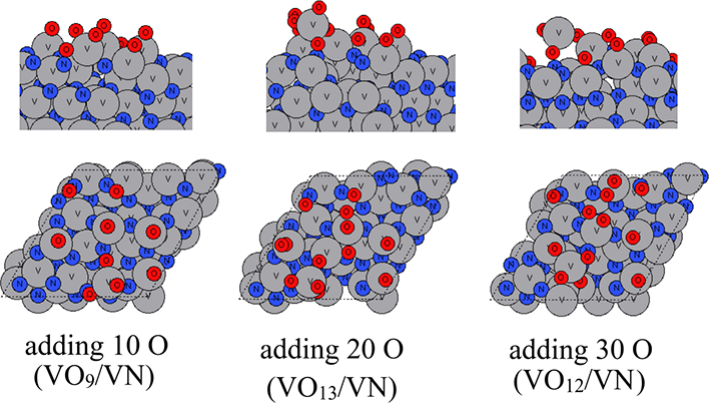
Atomic configurations after aiMD of ZrN surface oxidation on the VN (111) surface at 873 K. V atoms and N atoms are presented in gray and dark blue colors, respectively, and oxygen atoms are presented in red color.

If we increase the temperature to 363 K, then a similar partially oxidized VO_*x*_ is formed on top of VN (111) substrates. More V atoms are shifted upward. However, after adding 30 atoms of a V atom in the aiMD, although there are plenty of available surface V and N atoms, the newly added oxygen species tend to bind to surface oxygen to form O_2_ at the partially oxidized surface, and the resulting structure includes a partially oxidized V_8_O_18_ on top of the VN (111) substrate.

If we increase the temperature to 873 K, even at such a high temperature, the O species do not migrate into bulk VN, which is contrary to the ZrN (100) and ZrN (110) surfaces. Instead, O species induce an outward migration of surface V atoms to form a partial layer of oxidized VO_*x*_ on the surface, and this appears to block any subsequent oxidation process, resulting in V_5_O_12_ on top of the VN (111) substrate. With much less oxygen penetration observed for VN oxidation, the original VN surface slab is much less distorted.

#### 3.3.1 On the Difference between the Oxidation Processes of ZrN and VN Thin Films

Table 1 summarizes the formation of MO_*x*_/MN (M = Zr and V) at selected temperatures after aiMD calculations. With increasing temperatures, ZrN, in particular, the (110) surface, could be fully oxidized and form a thick surface ZrO_*x*_ layer, while the (100) surface would show a thinner ZrO_*x*_ layer. In contrast, even at higher temperatures, VN yields only partially oxidized, single layer VO_*x*_ islands on top of VN (111). We do not see the migration of the O species to bulk VN (111), but the migration of the O species into bulk ZrN (110) and (100) is enhanced with increasing temperature.

**Table 1:** Summary of the Formation of MO_*x*_/MN (M = Zr, V) at Selected Temperatures after aiMD Calculations^a^

material	surface	295 K	363 K	873 K	1023 K
ZrN	ZrN (100)	ZrO_8_/ZrN (0.89 ML)	ZrO_12_/ZrN (1.33 ML)	-	ZrO_28_/ZrN (3.11 ML)
	ZrN (110)	ZrO_18_/ZrN (2 ML)	ZrO_20_/ZrN (2.22 ML)	-	ZrO_32_/ZrN (3.56 ML)
VN	VN (111)	VO_11_/VN (0.68 ML)	VO_18_/VN (1.12 ML)	VO_12_/VN (0.75 ML)	-

a The coverage in parentheses indicates the concentration of O species per supercell.

This observation is consistent with the detailed compositional analysis in our previous publication on the MOCVD growth of ZrN thin films, where 1023 K is the optimized deposition temperature on Si(100), showing only a (200) reflex in the XRD results (Figure 11 a). The composition determined from RBS in combination with NRA (Table 2) yields specific concentrations of Zr, N, O, and C in the films as follows: 31.1 at. % (Zr), 35.7 at. % (N), 19 at. % (O), and 14.3 at. % (C). At lower deposition temperatures of 923 and 823 K, the amount of C and O contamination increases significantly. We excluded the significant oxygen incorporation during the deposition process, as there is no crystalline ZrO_2_ reflex in the XRD pattern. We attribute the O contamination to the postdeposition oxygen incorporation during the sample handling under ambient conditions. This DFT study has confirmed that the ZrN thin film could be easily oxidized at 363 K, and this oxidation process is self-limiting, which is in accordance with the experimental findings.

**Figure 11:**
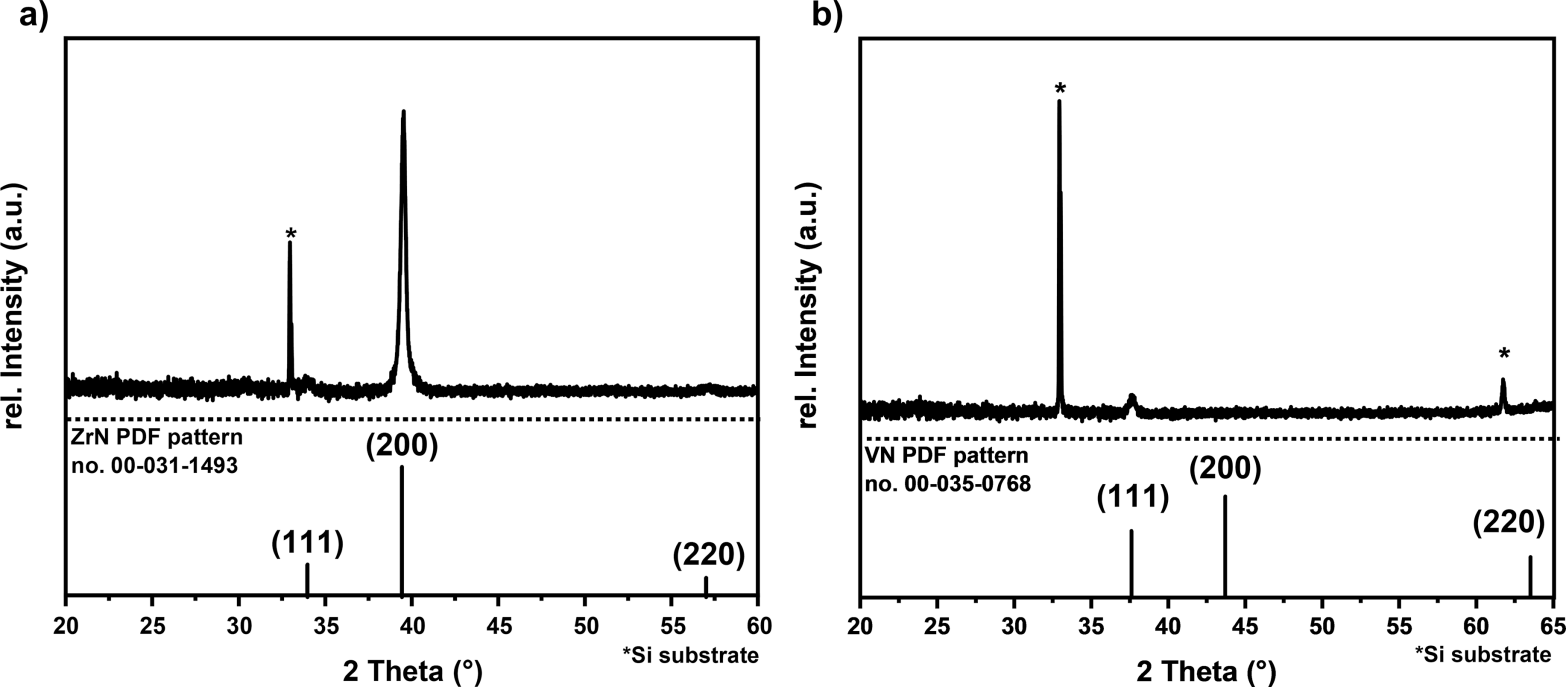
(a) XRD pattern of MOCVD grown ZrN on Si; reproduced from ref 29. (b) XRD pattern of MOCVD grown VN on Si. The XRD reference patterns of ZrN from PDF pattern no. 00–31–1493 ^41^ and VN from PDF pattern no. 00-035-0768.^42^

Available under a CC-BY license. Copyright 2024 Glauber et al.

**Table 2:** ZrN and VN Thin Film Compositions Derived from RBS/NRA Measurements

material	Zr^a^ (at. %)	N^a^ (at. %)	M/N	O^a^ (at. %)	C^a^ (at. %)
ZrN	31.1	35.7	0.87	19.0	14.3
VN	45.3	54.2	0.84	0.1	0.4

a For all concentration values, an error of ±2 at. % can be considered.

The experimentally grown unoptimized VN (111) thin films on Si(100) at 823 K, for which the XRD shown in Figure 11 b displays a small (111) reflex, have a bulk composition (RBS/NRA) of V, N, O, and C of 45.3 at. %, 54.2 at. %, 0.1 at. %, and 0.4 at%, respectively, indicating very little oxygen incorporation. Our subsequent experimental paper will present results for the optimized MOCVD of VN films for NRR, but this initial result clearly shows a difference between the oxidation of ZrN and VN that is consistent with the aiMD findings. The measured amount of oxygen (Table 2) in the thin films can be attributed to postdeposition oxidation under ambient conditions.

The surface oxidation of ZrN films is also clearly visible in X-ray photoelectron spectroscopy (XPS) analysis, which is shown in Figure 12 for ZrN after MOCVD and exposure to ambient conditions. The ZrN surface is largely dominated by contributions of the oxide components z3 and o1 and o2 in the high-resolution Zr 3d and O 1s spectra, respectively, while components belonging to nitrides (z1 and n2) and oxynitrides (z2 and n1) are diminished in the Zr 3d and N 1s spectra. After sputtering with Ar^+^ (2 keV, 60 s) to remove the top surface layer (in our previous study, a sputtering rate of 4 nm min^–1^ was determined^28^), the oxynitrides and to some extent also the nitride components become more pronounced. Nevertheless, there remains a strong contribution from oxide components, consistent with DFT aiMD observation of oxygen diffusion into the bulk of ZrN. Detailed depth profiling in our previous study showed a remaining oxygen content of around 25 atom % in the bulk (roughly 76 nm depth) and zirconium and nitrogen concentrations of 50 and 15 at. %, respectively.

**Figure 12:**
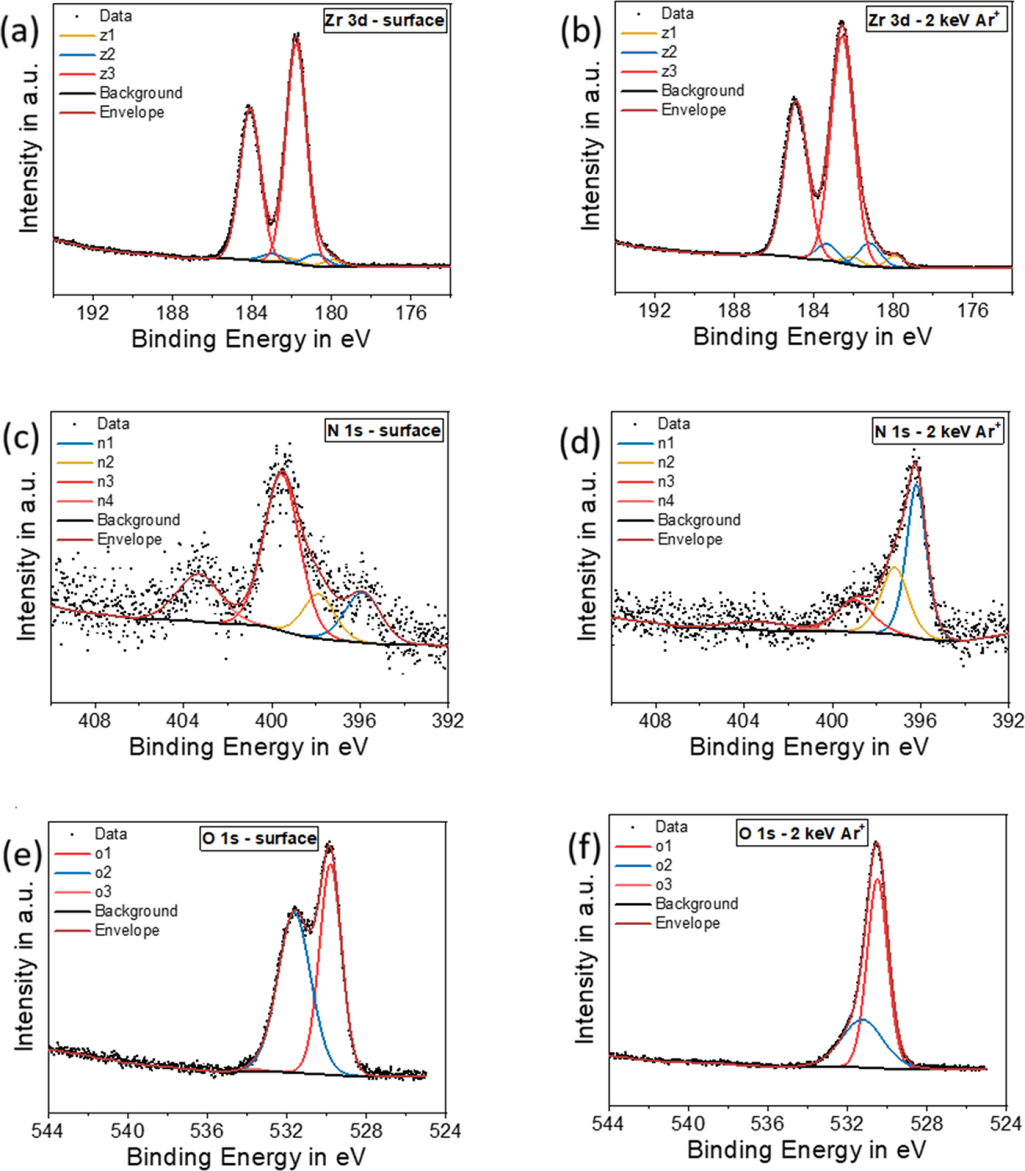
XPS spectra of ZrN thin films deposited on Si by using MOCVD. (a) Zr 3d from the surface layer. (b) Zr 3d after sputtering. (c) N 1s from the surface layer. (d) N 1s after sputtering. (e) O 1s from the surface layer. (f) O 1s after sputtering. Reproduced from ref 29.

Available under a CC-BY license. Copyright 2024 Glauber et al.

For the VN thin films, this DFT study has found that VN thin films are not easily oxidized even at higher temperatures. VO_*x*_ tends to form clusters on top of the VN (111) substrate. We attribute this significant difference in the oxidation process into several factors: (1) in terms of bond dissociation energy,^43^ the V–N bond (452 kJ/mol) is stronger than Zr–N (339 kJ/mol), whereas the V–O bond (645 kJ/mol) is weaker than the Zr–O bond (776 kJ/mol); this means that oxygen can more readily replace nitrogen, facilitating faster oxidation of ZrN compared to VN. (2) ZrO_2_ is more thermodynamically stable than V_2_O_5_, meaning that Zr has a much stronger tendency to bond with oxygen and form a stable oxide layer. The role of nitride oxidation on the NRR chemistry and the impact of the differences between ZrN and VN oxidation as well as differences between ZrN surface facets are the subject of ongoing work on these materials.

## 4 Conclusions

We simulated the oxidation process of ZrN and VN thin films at ambient conditions using aiMD methods at selected thin film growth temperatures of 295, 363, 873, and 1023 K. For ZrN thin films, we investigated two representative facets of ZrN (100) and ZrN (110), where ZrN (100) tends to form oxynitrides at low temperatures of 295 and 363 K and tends to form a ZrO_*x*_ layer at a high temperature of 1023 K. For ZrN (110), the surface region tends to form separate ZrO_*x*_ layers at all studied temperatures. For VN thin films, we investigated one representative facet of VN (111), where VO_*x*_ clusters are formed, and no significant migration of the O species into bulk VN is observed. On the other hand, for ZrN (100) and ZrN (110), the higher the temperature, the more oxygen species migrate into bulk ZrN. This is in accordance with the experimental composition analysis results where the O contents are 19 atom % for ZrN thin films. We conclude that VN is less oxidized than ZrN at ambient conditions because VN forms a less stable, sometimes volatile oxide layer, whereas ZrN has a stronger tendency to form a stable, protective ZrO_2_ layer, promoting more complete oxidation at higher temperatures. Currently, ongoing process optimization of MOCVD of VN thin films hints that the theoretically obtained results match with the experimental analysis. The roles of the oxynitrides of ZrO_*x*_N_*y*_/ZrN and VO_*x*_N_*y*_/VN found both experimentally and in these simulations are under investigation for their NRR properties.
